# Bridging the Gap in Cryopreservation: A Review of Cryoprotectant Innovation and Global Market Trends

**DOI:** 10.3390/bioengineering13050557

**Published:** 2026-05-15

**Authors:** Shankar Shanmuga Sundaram, Quang Bach Le, Deepak Choudhury

**Affiliations:** 1 Bioengineering and Automation (BE&A), Bioprocessing Technology Institute (BTI), Agency for Science, Technology, and Research (A*STAR), Singapore 138669, Singapore; e1121537@u.nus.edu (S.S.S.); quang_bach_le@a-star.edu.sg (Q.B.L.); 2 Department of Food Science and Technology, National University of Singapore, Singapore 117542, Singapore

**Keywords:** cryopreservation, cryoprotective agents, CPAs, cell therapy, tissue therapy, tissue engineering, tissue regeneration

## Abstract

Cryopreservation integrates technologies that enable the long-term preservation of biological materials at extremely low temperatures. Since the serendipitous discovery by Audrey Smith and Christopher Polge that glycerol enhances the survival of frozen chicken sperm, the field has advanced rapidly and found applications across cell therapy, biobanking, and tissue engineering. This concise review provides an overview of cryopreservation principles and the current commercial landscape in cell freezing media, highlighting key players, challenges and recent developments in tissue and small-organ preservation.

## 1. Importance of Cryopreservation

Historically, it has been known that low temperatures can increase the shelf life of biological matters. Archeological findings indicate the use of icehouses to preserve food and wine in the Mesopotamia region as early as 2000 BC [1]. In modern times, cryopreservation is used for preserving living systems. The first effective trace of a cryoprotective agent was accidentally discovered by Polge in the 1940s. Someone swapped Polge’s fructose solution with Meyer’s egg albumen, which contained a mixture of egg white and glycerol. This substitution preserved the sperms’ fertilizing capacity, leading to the first successful fertilization and hatching of chicks from cryopreserved semen in 1949 [2].

Today, cryopreservation is a well-established method for long-term storage of cells and tissues, playing a critical role in biological research and medicine, enabling indispensable applications such as storing red blood cells in blood banks and maintaining donor tissues in tissue repositories. At extremely low temperatures, biological systems lose the thermal energy required for molecular and metabolic activity, slowing cellular and metabolic processes, keeping the specimen in a state of suspended animation hence making preservation possible [3].

**Figure 1:**
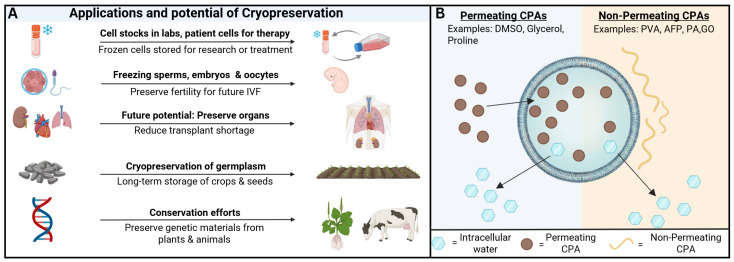
Applications and working mechanism of cryopreservation. (**A**) Current applications of cryopreservation across different sectors and the emerging potential in organ banking. (**B**) Schematic of the mechanism of permeating cryoprotectant agents (CPAs) and non-permeating CPAs. Abbreviations: in vitro fertilization (IVF); cryoprotective agent (CPA); dimethyl sulfoxide (DMSO); polyvinyl alcohol (PVA); antifreeze protein (AFP); polyampholyte (PA); and graphene oxide (GO).

Cryopreservation has a wide range of applications in three main industries: healthcare, agriculture and conservation [4] (Figure 1A). In biomedical research, nearly all laboratories maintain frozen cell stocks to prevent phenotypic drift and preserve rare and valuable samples [5]. In reproductive medicine, freezing oocytes, sperm, and embryos have transformed in vitro fertilization, allowing individuals to preserve fertility even before treatments like chemotherapy. Cryopreservation also underpins cell-based therapies like stem cell therapy and adoptive cell treatments for cancer by enabling safe transport and storage of patient-derived cells [6]. Emerging techniques for tissue and organ preservation may one day overcome the transplant shortage [7]. Applications of cryopreservation in the healthcare industry may also extend to the cosmetic and dermatology industry, such as preserving skin cells to use in cosmetic and dermatological treatments. In the agriculture sector, cryopreservation of germplasm enables long-term storage of vegetatively propagated crops, recalcitrant and intermediate seeds, and orthodox seeds with limited longevity. Cryopreservation also supports advanced applications such as cryoselection and cryotherapy, where freezing selectively eliminates viruses or unwanted cell populations [8]. Lastly, cryopreservation plays an important role in conservation efforts and breeding programs for preserving genetic material from endangered plant and animal species [9].

This review focuses on the cryopreservation of mammalian cells, driven by the rapid growth of cell therapy as one of the most innovative and fastest-expanding areas in biomedical research. Recent approvals of multiple cancer cell therapies highlight the need for robust cryopreservation strategies in ensuring safe, scalable, and effective treatments [10]. The aim of this review is to explore the underlying mechanism of cryopreservation and the various classes of cryoprotectants, while providing a comprehensive analysis of the current cryopreservation market and its future trajectory.

## 2. Mechanism of Cryopreservation

The normothermic range for mammalian cells is 36 °C and 38 °C and exposure to lower temperatures can lead to hypothermia. Nevertheless, ultra-low temperatures themselves are not inherently pathogenic, but the survival is primarily determined by events occurring during freezing and thawing, particularly at the water–ice transition. Biological tissues are predominantly aqueous, with water constituting approximately 80% of the total mass [11]. During freezing, the drop in temperatures below the freezing point thermally induces a phrase transition of the water molecules. They are arranged into an ordered crystalline lattice accompanied by volumetric expansion. The phase change of water in both intracellular and extracellular compartments leads to cryoinjury. Cryoinjury includes cellular dehydration, osmotic shock, intracellular and extracellular structural damage and oxidative stress. Osmotic shock refers to the sudden change in solute concentration leading to osmotic pressure imbalance.

As the temperature drops below the freezing point, extracellular aqueous solution begins freezing first, causing water concentration to drop and extracellular matrix concentration to rise. This creates an osmotic gradient between the cell and extracellular component as water rapidly leaves the cell, leading to osmotic shock, shrinkage and dehydration of the cells. Limited cellular dehydration is beneficial during the freezing process as it reduces intracellular ice formation (IIF). However, excessive dehydration can lead to irreversible damage to the cell [12]. In addition, extracellular ice formation damages the cell membrane by puncturing it or disrupting its structure, causing structural damage. Furthermore, nucleation of ice is an exothermic process. The release of latent heat imposes additional damage to surrounding cells [13]. Lastly, decreased enzymatic activity of the cells due to lower temperatures causes adenosine triphosphate (ATP) deficiency which leads to accumulation of calcium ions and oxidative stress [14].

Likewise, the thawing process introduces distinct biophysical challenges. During warming, the dehydrated cells are exposed to large volumes of water or buffer solutions. This generates a steep osmotic gradient causing a rapid rise in water across the cell membrane, leading to cell swelling and lysis [6]. Cells are also susceptible to ice recrystallization, particularly just below the freezing point [12]. Smaller ice crystals aggregate into larger ones, exerting mechanical damage and osmotic stress onto the cells. These factors undermine the survivability of cells.

The detrimental effects of freezing and thawing can be mitigated using cryoprotective agents (CPAs). CPAs inhibit ice formation when cooled to cryogenic temperatures which reduce cellular dehydration and osmotic stress while inhibiting ice nucleation and crystal growth (Figure 1B) [11]. However, CPAs are inherently bad for cells as excessive exposure can lead to cytotoxicity and osmotic imbalance during loading and removal. Cytotoxicity can alter cellular function and structure and even persist inside the cells after multiple washes, leading to unintended downstream effects [15]. CPA toxicity is generally categorized as specific or non-specific toxicity. Specific toxicity refers to cellular damage induced by a mechanism unique to a particular CPA, such as specific enzyme inhibition or metabolization, while non-specific toxicity refers to the damage from properties common to all CPAs. The toxic effects of CPAs rise with temperature. This can be mitigated through loading ramp protocols where CPAs are added at increasing concentrations as the temperature of the sample drops. This can significantly improve the post-thaw viability [3].

Based on these principles, cryopreservation can be categorized primarily into slow (controlled-rate) freezing and vitrification (rapid freezing), with slow freezing being the most widely adopted. Emerging techniques such as high-pressure freezing, isochoric freezing, ultrasonic freezing and electro-freezing remain largely experimental [4,7]. The main distinction between slow freezing and vitrification lies in the cooling rate and concentration of CPAs. Slow freezing employs a gradual reduction in temperature, approximately of 1 °C/min, to −196 °C with minimal CPA addition while vitrification involves high concentrations of CPAs with extremely high cooling to achieve solidification through the formation of a nanocrystalline glass phase in the cells and tissues, bypassing the ice crystalline phase and preventing any ice formation [12].

While vitrification theoretically offers a superior preservation state by avoiding ice formation which is pathogenic to cells, its practical application remains limited compared to slow freezing. Although slow freezing is time-consuming, it is widely adopted as it complies with Good Manufacturing Practice (GMP) and is better suited for quantifiable and reproducible protocols. Vitrification requires high concentrations of CPAs and rapid cooling rates. High concentration of CPA is a concern, as it is cytotoxic. Additionally, rapid cooling and warming may induce mechanical fracturing of the glass in which the biological sample is embedded in [12]. However, there is no clear consensus on which method is superior, as the optimal approach depends on the specific application and context. Clinically, vitrification is commonly preferred for the preservation of oocytes and embryos [6].

Successful cryopreservation lies in optimal freezing protocols, composition and amount of CPA added, optimal cooling rates and equilibration protocols. Successful thawing can be achieved through rapid and uniform heating via optimal warming techniques. These parameters vary substantially across different cell types due to varying biochemical pathways, emphasizing the need for tailored cryopreservation strategies [16].

## 3. CPA Classification: Permeating vs. Non-Permeating Strategies

CPAs are broadly categorized into two groups: permeating or non-permeating (Figure 1B). Permeating CPAs are small, highly soluble molecules that diffuse into cells and replace intracellular water without excessively dehydrating the cell. They solidify at lower temperatures than water which reduces intracellular ice formation and thereby limits structural damage during freezing. Examples include dimethyl sulfoxide (DMSO), glycerol, ethylene glycol and proline, with DMSO being a standard agent for various cell types such as stem cells, animal cells, sperms, oocytes and embryos [6]. Non-permeating CPAs are long-chain polymers and remain outside the cell, increasing extracellular osmolarity to dehydrate the cell and prevent intracellular ice, while also suppressing extracellular ice crystallization on the cell membrane. They are often used during thawing by being added in stepwise to balance the osmotic pressure as the permeating CPAs are removed. Non-permeating CPAs consist of sugars and polymers such as polyvinyl alcohol (PVA), antifreeze protein (AFP), polyampholyte (PA), and graphene oxide (GO). Among them, PVA is typically combined with other CPAs, AFP has applications in food preservation, agriculture, and cryomedicine, PA is compatible with multiple cell types, and GO has shown potential in sperm cell cryopreservation [6].

## 4. Novel Cryoprotectant Development

Future growth in this field will depend on the development of cell- and organ-specific, low-toxic, perfusable CPA. One such innovation includes a glycerol and DMSO-free amino acid-based CPA consisting of two different amino acids: phenylalanine and proline managed to preserve sheep red blood cells (RBCs) successfully with a viability of 85% post recovery [16]. Another possible direction for next-generation cryoprotectants is the use of antifreeze proteins (AFPs). These naturally occurring molecules, found in cold-adapted organisms such as insects, fish and plants, enable survival under sub-zero conditions. However, they cannot be translated to mammalian cells due to immunogenicity [17,18]. Synthetic AFPs have shown promise as engineered cryoprotectants. A systematic review evaluating AFP performance over the past three decades reported that, across 66 mammalian-cell experiments, 71.4% demonstrated improved post-thaw survival [18,19]. Similarly, in another study, low immunogenic antifreeze peptides were developed, resulting in 81.32% post-thaw viability in mouse cells [17]. Lastly, natural deep eutectic solvents (NADES) such as quaternary ammonium salts, amino acids, and sugars could be a green, non-toxic alternative to DMSO. In a study comparing two NADES and DMSO solvents, the viability of A549 cells post-thaw was higher among NADES than DMSO under specific conditions [20]. Along with CPA development, advanced cooling and warming technologies such as nanowarming and inductive heating could expedite the research towards organ freezing [9].

## 5. Commercial Freezing Media Market

The global cell cryopreservation market was valued at USD 3.38 billion in 2024 and is expected to grow to USD 8.86 billion by 2033, reflecting an annual growth rate of 11.40% from 2025 to 2033. North America had the largest share in the cell cryopreservation market of 40.46% with a fast-growing rate observed in the Asian market [21].

The cell cryopreservation market is expanding primarily due to two key drivers: growing demand for fertility preservation and rapid advancements in cell and gene therapies. Increased use of Assisted Reproductive Technology (ART), rising infertility rates, and lifestyle-related delays in parenthood have intensified the need to preserve gametes and embryos, while the expansion of fertility clinics and supportive policies further contribute to this trend. At the same time, the wider adoption of cell and gene therapies and stem-cell-based treatments has created strong demand for reliable freezing media and cold-chain systems to maintain cell viability. Investments in biobanking, fertility programs, and clinical trials further reinforce this growth, collectively driving ongoing development of cryopreservation technologies and infrastructure [21]. Specifically, the fastest growth in the Asia Pacific region is fueled by growth in regional stem cell banking networks and government backed regenerative medicine programs. In particular, China, Japan, and South Korea have been instrumental in this growth, investing in large-scale cryogenic facilities integrated with advanced data management systems to support national cell therapy initiatives and clinical research programs [22].

**Figure 2:**
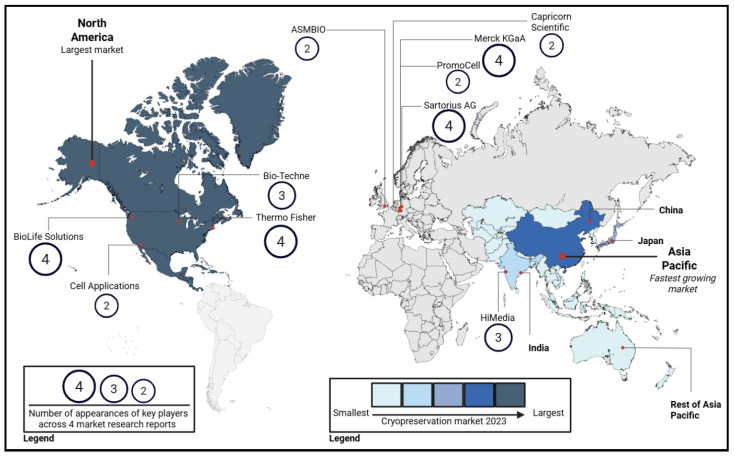
Global map showing cryopreservation market. The legend on the left represents the number of appearances of key players in the cryopreservation media market according to four independent market research reports [21,23,24,25,26]. The legend on the right compares the size of the cryopreservation market across North America and Asia Pacific only.

The cell freezing media market is primarily driven by a group of major global manufacturers that consistently appear across multiple independent market research reports. *Thermo Fisher*, *Merck KGaA*, *Sartorius AG*, and *BioLife Solutions* were identified in all four reports, indicating their strong market dominance. *Bio-Techne* and *HiMedia* were each featured in three of the four reports, further highlighting their significant presence in the sector (Figure 2). The following section provides a detailed overview of the freezing media offered by these six key companies [21,23,24,25] (Table 1). It is important to note that cryopreservation specifically for in vitro fertilization (IVF) represents a distinct market segment on its own. In this specialized field, soybean-derived extracts, specifically lecithin, have been increasingly studied as a chemically defined, plant-based alternative to traditional egg yolk-based extenders. This transition aims to mitigate the biosafety risks and batch-to-batch variability associated with animal-derived components during sperm freezing. According to *Future Market Insights*, the top three companies in this space are *Vitrolife AB*, *Merck KGaA*, and *CooperSurgical, Inc.* [26]. However, *Merck KGaA* focuses primarily on fertility drugs and devices [27]. We therefore examine the cryopreservation media developed by *Vitrolife* and *CooperSurgical, Inc.* only (Table 1).

## 6. Current Progress in Tissue and Organ Cryopreservation

Although cryopreservation is primarily used for cells, recent advances have enabled successful preservation of small tissues and even organs. Small tissue cryopreservation is becoming increasingly standardized [28,29]. For example, Ishizaki et al. developed a cryoprotectant containing an artificial zwitterion combined with DMSO, effectively preserving cell-spheroid models of mouse and human tumor tissues [30]. Similarly, Xue et al. cryopreserved brain tissue and neural organoids using a formulation of methylcellulose, PEG, DMSO, and Y27632 (MEDY), maintaining both neural cytoarchitecture and functional activity [31].

Whole-organ cryopreservation remains significantly more challenging than cells or small tissues, but recent advances have shown promising progress. Han et al. demonstrated successful cryopreservation kidney in a rat model using vitrification, forming an ice-free glass like state [32]. The challenge occurs during thawing where ice recrystallization can damage the tissue. To overcome this, the study used a novel nanowarming technique in which magnetic fields were employed to heat the nanoparticles within the organ vasculature, enabling rapid and uniform warming. The kidney was cryopreserved for 100 days, then thawed and transplanted, restoring function and sustaining life in the recipient rat. Ozgur et al. optimized a partial high-subzero storage protocol at −15 °C that enables 10-day storage of rat livers. This was achieved through increased concentration of polyethylene glycol (PEG) and bovine serum albumin. Optimal preservation and functional post-thaw liver was observed through various parameters [33]. Notably, this protocol extended liver preservation to up to 10 days, outperforming the current gold standard static cold storage (SCS) which can preserve a liver for 8–10 h [33,34]. This study was conducted on small animals and replicating it on larger animals may bring about other issues such as eddy current heating causing non-uniform power deposition, non-uniform distribution of nanoparticles and CPA diffusion [35].

Wang et al. reported two remarkable clinical cases in which amputated fingers were cryopreserved and later successfully replanted [36]. In both cases, the patients with the amputated finger refused replantation initially and the amputated finger was cryopreserved in a solution consisting of 10% fetal bovine serum, 12% DMSO, and 78% RPMI-1640 medium. One finger was cryopreserved for 81 days while the other was preserved for 5 days. They used a multi-stage freezing protocol where the sample was maintained at 4 °C for 15 h in a sealed, sterile polyethylene bag, followed by stepwise cooling at −20 °C for 4 h and −80 °C for 24 h, before final transfer to liquid nitrogen (−196 °C). Subsequently, they returned to the hospital requesting for a replantation and their cryopreserved fingers were utilized to replant them. In both situations, the surgery led to satisfactory appearance and function of the finger.

Despite the success in organ cryopreservation discussed earlier, to date, clinical translation of cryopreservation has been predominantly successful for tissues with low metabolic demands or relatively simple structures such as bones, adipose tissue, skin, testicular tissue, ovarian tissue, amniotic membrane, heart valves and blood. However, significant technology plateau exists when attempting to cryopreserve vital, complex organs. For instance, there are no established protocols for long-term storage of heart and there are only limited results reported in the cryopreservation of lungs [37]. The lack of succession is due to various factors such as size, complex distribution of cells, and varying protocols for each organ.

On the organ scale, there are many issues that the current state of cryotechnology cannot solve. Beyond the challenges faced in a cellular level, which includes osmotic stress, mechanical damage, CPA toxicity and oxidative damage, organs are subjected to non-uniform CPA penetration, heterogeneous cooling rates and thermomechanical stress [3,6,29,38,39]. These factors limit the scalability of cryopreservation from a cellular level to the organ level. First, heat and mass transfer in organs are significantly more complex than in cell suspensions. The loading and removal of CPAs, as well as water distribution, are the key considerations [40]. At an organ level, increased size, various types and arrangement of cells and various interactions between cells lead to non-uniform distribution of CPAs on the surface and interior of the organ [40,41]. Similarly, heat transfer is also a challenge which is limited by macroscopic volume and thermal conductivity of the organ, compromising uniform cooling and warming. Uneven heat distribution can impose mechanical stress and rupture the tissue or organ [42]. This is worsened by the formation of ice crystals within the cracks during the rewarming process [43].

**Table 1:** Summary of the key commercial reagents sold by the companies listed above.

Company	Medium Name	Composition	Target Cell Types	Grade
*ThermoFisher*	*(CTS)Synth-a-Freeze* [44]	10% DMSO, antibiotic-free, serum-free, protein-free	Various mammalian cells (except melanocytes)	RUO/cGMP
	*Recovery Cell Culture Freezing Medium*	10% DMSO, high glucose, phenol red, with FBS	Mammalian cells	RUO
*Sartorius AG*	*NutriFreez D5/D10* [45]	5%/10% DMSO, animal component-free, serum-free, protein-free	Various	cGMP
*BioLife* *Solutions*	*CryoStor CS5/CS10* [46]	5%/10% DMSO, serum-free, protein-free	Various	cGMP, USP
	*BloodStor 27/55/100*	27%/55%/100% DMSO in saline, animal component-free, serum-free	Stem cells, hematopoietic stem and progenitor cells	cGMP, USP
*Merck KGaA*	*C9249 CryoSOfree DMSO-free* [47]	DMSO-free, animal component-free	Various	RUO
	*C6295 Cell Freezing Medium*	8.7% DMSO, serum-free	Various	RUO
*Bio-Techne*	*CryoDefend-Cell Lines* [48]	Contain DMSO, protein-free	Multiple cell lines	RUO
	*StemXVivo® Serum-Free MSC Freezing Media*	10% DMSO, serum-free, antibiotic-free	Human/mouse/rat MSC	RUO
*HIMEDIA*	*CryoXL™ Cell Freezing Medium* [49]	DMSO, FBS	Mammalian cells	RUO
*Vitrolife*	*SpermFreeze Solution* [50]	MOPS, HSA (human serum albumin), glycerol, cholesterol	Human sperm	Regulatory approved
	*RapidVit & RapidWarm*	MOPS, HSA, cryoprotectants	Oocytes, embryos of various stages	Regulatory approved
*Cooper* *Surgical*	*Quinn’s Advantage Sperm Freezing* [51]	HEPES, HSA, phenol red, gentamicin	Human sperm	
	*Quinn’s Advantage Embryo Thaw Kit*	HSA	Embryos and blastocysts	Regulatory approved
	*SAGE Vitrification Solutions*	NA	Oocyte and embryo	Regulatory approved
	*Global Blastocyst Fast Freeze Thawing Kit*	HSA, DMSO-free	Human blastocyst	
	*Global DMSO Blastocyst Vitrification Kit*	DMSO, HEPES, HSA	Human blastocysts	
	*Sperm Freezing Medium/CryoSperm*	Insulin, sucrose, egg yolk-free, HSA-free, HEPES-free	Human sperm	
	*Embryo Freezing/Thawing Pack*	HSA, propylene glycol, sucrose	Human zygotes and cleavage stage embryos	
	*MediCult Vitrification Cooling/Warming*	DMSO-free, HEPES, HSA	Human oocytes, embryos	

FBS: fetal bovine serum, HSA: human serum albumin, MOPS: 3-(N-morpholino) propane sulfonic acid buffer, RUO: for research use only, cGMP: current good manufacturing practice, USP: United States Pharmacopeia grade.

## 7. Regulatory Challenges to Commercialization

Cryopreservation media fall under the broader category of ancillary materials (AMs) or excipients. It is considered as AM when reagents or components are used in the production of cell therapy products, but they are not meant to remain in the final therapeutic product [52]. It is considered as excipients when residual components of the media, such as DMSO, are left behind in the final product. Hence, the use of CPAs in clinical trial cell and tissue products is governed through ancillary material and excipient frameworks rather than CPA-specific regulations [53].

Title 21 of the Code of Federal Regulations, Part 312 (21 CFR 312) governs Investigational New Drug (IND) applications. Under this framework, CPAs used during manufacturing but not present in the final product, as well as CPA components that remain in the final administered product (e.g., residual DMSO or human serum albumin), must be appropriately justified in the IND. Such materials are subject to the requirements of 21 CFR 312.7(b) and should comply with applicable United States Pharmacopeia–National Formulary (USP–NF) standards [53]. Moreover, FDA’s CMC guidelines recommend sponsors to define impurities (e.g., DMSO), have tests in place to measure them, provide acceptance limits and remove them [54]. With respect to USP <1043> Ancillary Materials for Cell, Gene and Tissue-Engineered Products, ancillary materials need to undergo risk assessment as stated [55]. Additionally, biological products such as FBS and HSA (human serum albumin) must comply with the FDA’s guidance, Considerations for Use of Human- and Animal-Derived Materials, which could be shown through documentation such as providing test results, screening, traceability, certificates of analysis (COAs) and Adventitious Agents Safety Evaluation [56]. Lastly, under 21 CFR 210/211, it states that components of the final drug product must adhere to the cGMP standards and samples from each lot of components will be tested or examined before being released for use by the quality control unit [57,58].

## 8. The Gaps in Current Cryopreservation Media Market and Future Potential

The current cryopreservation media market is built almost entirely around single cells such as sperm, oocytes, stem cells, and other cell-based products. These media are optimized for microscopic scale freezing, where CPA diffusion, cooling rates, and warming profiles are tightly controlled. As a result, commercial products effectively serve fertility clinics, research laboratories, and biobanks, but they do not address the far larger unmet demand in organ preservation and transplantation [15,29,39].

The core market gap lies in the inability to scale existing formulations and protocols to large, complex tissues or organs [3]. At the organ scale, technical failures become unavoidable: non-uniform CPA penetration, heat and mass transfer limitations, heterogeneous cooling rates, thermomechanical stress, CPA toxicity at required concentrations, and ice recrystallization during thawing [3,6,29,38,39]. These issues reflect not only scientific challenges but also the absence of commercially viable, regulatory-ready products designed for organ-level preservation. This gap has a direct economic and clinical impact. High organ rejection rates and the discard of thousands of abdominal and thoracic organs have imposed substantial costs on healthcare systems and limited the number of transplantable organs.

The integration of artificial intelligence (AI) and machine learning (ML) in cryopreservation research could represent a transformative frontier in addressing these gaps by shifting from trial-and-error to data-driven experimentation. In novel CPA development, ML models using molecular simulations have been used to analyze vast chemical libraries to identify potent small-molecule ice recrystallization inhibitors [59]. For protocol optimization, AI-driven optimization algorithms, such as Differential Evolution (DE) and Bayesian approaches, have been employed to tailor multicomponent freezing protocols and cooling rates for specific cell types like mesenchymal stem cells and Jurkat cells [60]. Furthermore, AI is revolutionizing quality control (QC) and real-time monitoring through machine vision and predictive modeling; convolutional neural networks (CNNs) have been utilized to dynamically track critical quality attributes (CQAs) such as cell morphology and genetic integrity during the biomanufacturing process [61]. These future real-time monitoring systems could ensure the high-fidelity preservation required for clinical-scale large-organ banking. An effective organ preservation technology would fundamentally expand the market by enabling organ banking, improving logistics, reducing time pressure on transplant teams, increasing utilization rates, and allowing more thorough donor–recipient matching, which in turn reduces immune rejection risk and downstream clinical complications, representing an untapped commercial opportunity [39].

However, integration of AI into cryopreservation is hampered by several regulatory hurdles. The opaque nature of machine learning algorithms often conflicts with the transparency required by the FDA and other agencies for clinical validation. To address this issue in the drug development sector, the European Medicines Agency (EMA) and the FDA recently established the guiding principles for good AI practices which mandate rigorous data governances and documentation [62]. Similarly, successful adoption of AI and ML in cryopreservation will require proper regulations such as good machine learning practices to enforce data standardization and ethical, transparent use of these data algorithms.

## 9. Conclusions

The importance of cryopreservation has increased markedly with recent advances in cell and gene therapy, genetic engineering, and tissue engineering. As a foundational platform across biomedical and biomanufacturing sectors, it is positioned for exponential market growth as global demand for drug research, scalable cell therapies, gene therapies, engineered tissues, and advanced biotherapeutics accelerates. Despite its prevalence, the cryopreservation sector is still constrained by limited understanding of its mechanism, especially regarding larger, complex tissues. Progress requires a collaborative, interdisciplinary approach that bridges biophysics, molecular biology, thermodynamics and other relevant study fields. Coordinated efforts among research communities, funding agencies, industry partners, regulatory stakeholders and integration of artificial intelligence and machine learning are vital to expedite progress and scale up cryopreservation.

## Data Availability

Not applicable.
